# SSD-12 somatic symptom disorder scale: replication of validation and introduction of a parent version SSD-12-P

**DOI:** 10.3389/fpsyg.2026.1816120

**Published:** 2026-05-14

**Authors:** Marlene Abel, Stefanie M. Jungmann

**Affiliations:** 1 Department of Clinical Psychology, Psychotherapy, and Experimental Psychopathology, Johannes Gutenberg-University Mainz, Mainz, Germany; 2 Department of Clinical Psychology and Psychotherapy for Children and Adolescents, Johannes Gutenberg-University Mainz, Mainz, Germany

**Keywords:** psychometric, reliability, replication study, self-report, somatic symptom disorder, SSD-12, SSD-12-P, validation

## Abstract

According to DSM-5, Somatic Symptom Disorder (SSD) is characterized by distressing somatic symptoms with accompanying excessive thoughts, feelings, or behaviors. SSD is highly prevalent in the adult population and imposes significant clinical and societal burden. Early identification and standardized assessments are highly relevant, particularly for effective treatment. The SSD-12 is a validated self-report measure focused on the B-criteria of SSD; however, analogues parent-reported instruments for concerns about children’s somatic symptoms are lacking. Moreover, independent replications of the SSD-12’s factor structure and psychometric performance in new samples remain limited, which is essential to support its routine screening use. To address these gaps, this study (1) re-examined the factor and psychometric quality of the SSD-12 in adults and (2) validated a newly developed parent version (SSD-12-P) assessing parental stress triggered by child somatic symptoms. In an online study, 564 adults and a subsample of 144 parents completed the respective instruments. Confirmatory factor analyses supported the established three-factor structure (behavioral, cognitive, affective) for both, the SSD-12 and SSD-12-P, with strong factor loadings and high internal consistencies. SSD-12 and SSD-12-P demonstrated robust convergent validity via correlations with health anxiety (WI-7) and with each other. Findings support the SSD-12’s reliability for adult screening and introduce the SSD-12-P as a promising instrument to capture parental contributions to intergenerational somatic symptom burden, with implications for targeted interventions and future longitudinal research.

## 1 Introduction

Persistent somatic symptoms are common in the general adult population, with prevalence ranging from about 26 to 65% depending on age, gender (with higher rates in women), and assessment method (Beutel et al., 2020; Beutel et al., 2019; Kocalevent et al., 2013; Sperling et al., 2023). Unspecific somatic symptoms are highly prevalent in the adult general population. A cross-sectional study from 2019 examined the prevalence of somatic complaints in individuals aged between 40 and 80 using the PHQ questionnaire. The study found that 65% of the sample reported at least any mild somatic symptoms at the time of survey. Among women, approximately 70% exhibited at least mild symptoms, whereas about 59% of men reported the same. Further, they found a prevalence of somatization disorder of 9.3%. Here also, the rates are higher among women (15.6%) than for men (7.4%) (Beutel et al., 2019).

Regarding the prevalence of the categorical diagnosis of somatic symptom disorder (SSD) as defined in the Diagnostic and Statistical Manual of Mental Disorders (DSM-5, APA, 2013), research suggests a population-based prevalence between 5 and 17%, averaging around 13%. In primary care settings, around 8% of patients meet full SSD criteria (Huang et al., 2023; Kop et al., 2019; Lehmann et al., 2022; Löwe et al., 2022).

Early detection and accurate diagnosis are crucial, particularly given the high prevalence and burden for affected individuals. Accordingly, it is necessary to have reliable and valid screening and diagnostic assessment instruments to assess SSD in the general population. In the DSM-5 (APA, 2013), the former somatoform disorders were restructured and redefined to SSD. SSD is characterized by the presence of at least one distressing somatic symptom (A criterion), accompanied by at least one psychological feature—such as persistent thoughts about the seriousness of the symptoms, persistently high levels of anxiety about health and/or excessive of time/energy devoted to these symptoms (B criterion), and a typical duration of 6 months (C criterion). In line with this reconceptualization, a corresponding self-report questionnaire was developed.

The SSD-12 is a validated screening instrument to assess the severity of the DSM-5 SSD -B-criterion (SSD-12; Toussaint et al., 2016). The SSD-12 primarily captures criterion B and can provide indications of the presence of SSD by assessing excessive *thoughts*, *feelings*, and *behaviors* related to somatic symptoms or associated health concerns. The 12 items closely reflect the DSM-5 classification system: (1) disproportionate and persistent thoughts about the seriousness of symptoms, (2) persistently high levels of anxiety about health or symptoms, (3) excessive time and energy spent on these symptoms or health concerns (Toussaint et al., 2016). A key feature of the SSD-12 is its differentiations of behavioral, cognitive, and affective components. In addition to the aim of screening, it is well suited for routine outcome monitoring to evaluate treatment response and to track changes in symptom-related impairment over time.

Somatic symptoms also play a major role within family systems. On the one hand, children and adolescents also show a relatively high prevalence of persistent somatic symptoms (Berntsson et al., 2001; Rask et al., 2009), and on the other hand, parents, especially those with own complaints or health anxiety, are concerned about their children’s somatic complaints (Ingeman et al., 2024). Up to one quarter of children and adolescents report recurrent, distressing somatic symptoms, such as headaches or abdominal pain (Berntsson et al., 2001; Rask et al., 2009). In particular, an increase in somatic symptoms can be observed in late childhood and early adolescence (Lieb et al., 2002). In an inpatient sample of youth aged 11–17 years (Geremek et al., 2024), approximately 60% reported indications of a somatoform disorder (DSM-IV terminology) in the past 6 months and about 45% in the past week.

Caregivers such as parents are often the first to notice and respond when children experience bodily symptoms (Elliott et al., 2020). They decide, for example, how to react to complaints, e.g., by regulating emotions or by taking concrete actions such as seeking medical advice. Parents may be particularly likely to worry about their children’s complaints and health when they themselves suffer from somatic symptoms or elevated health anxiety. This phenomenon, termed health anxiety by proxy, refers to excessive parental fears that their child may suffer from a serious illness that have been overlooked by health care professionals (Ingeman et al., 2024). In the present study, the construct of interest is not general parental burden associated with caring for a child with physical symptoms. Rather, the SSD-12-P is intended to operationalize the DSM-5 Somatic Symptom Disorder B-criteria in a by-proxy format. Accordingly, the scale assesses maladaptive cognitive, affective, and behavioral responses in the parent that are triggered by the child’s somatic signals, bodily processes, or physical complaints. The focus is on the parent’s own psychopathology and symptom-related distress, not on the child’s symptom severity and not on unspecific caregiving strain. Health anxiety by proxy is discussed in this paper because it is an empirically established related construct, but the present concept is broader and is anchored in the DSM-5 SSD framework, which subsumed earlier diagnostic entities such as hypochondriacal and somatization-related presentations.

Parental factors can play a significant role in the development and maintenance of psychological symptoms and disorders in children (Hosman et al., 2009). For example, parental concerns and behaviors may directly influence children’s symptom experiences and coping, and parent–child processes may also be reciprocal; for example, children observe and learn from how their parents respond to physical complaints (Clementi et al., 2019; Goodman and McGrath, 2003; Losin et al., 2017; Palermo et al., 2014; van Tilburg et al., 2015). Jungmann and Witthöft (2020) conducted a pilot study in which children completed an age-adapted version of the SSD-12 (SSD-CA), while parents answered a parent-adapted version (SSD-12-P). In this study this parent self-report measure of worry about children’s somatic symptoms (as parental stress by proxy) was estimated for the first time. They found a medium-to-strong correlation (*r* = 0.44, *p* ≤ 0.001) between the SSD-12-P and the SSD-CA scores. In line with this, Jungmann et al. (2022) found that the only significant predictor of parent-reported physical complaints in children was parent’s own physical complaints.

Understanding the parental perspective is essential not only for identifying risk factors and informing therapeutic interventions, but also for capturing the full extent of somatic symptom-related burden within the family system. Assessing parental cognition, emotional responses, and behavior provides a more comprehensive view of how somatic symptoms affect not only the child, but also broader family functioning. Supporting parents in managing their own responses may therefore be a crucial part in preventing the intergenerational transmission and maintenance of somatic symptom-related distress. However, to date, no validated parent self-report instrument is available to assess parental stress and impairment related to children’s somatic symptom (disorder) according to DSM-5. To address this gap, the second aim is to systematically validate the parent version of the SSD-12 (SSD-12-P) as a measure of the parent’s own SSD-related psychological burden by proxy, triggered by the child’s somatic symptoms.

Overall, the present study had the following objectives and hypotheses, which were achieved in two parts:

(1) Replication of the factor structure and psychometric quality of the SSD-12 in adults according to the results of Toussaint et al. (2016).

*Hypothesis 1*: posits that the three-factor structure can be replicated in the current sample.

*Hypothesis 2*: assumes that acceptable reliability and validity can be replicated in the current sample.

(2) Validation of the newly developed SSD-12-P for parents (Jungmann and Witthöft, 2020; SSD-12) as a parent self-report measure about their own stress through children’s somatic symptoms.

*Hypothesis 3*: The SSD-12-P is expected to demonstrate a three-factor structure (affective, cognitive, and behavioral), consistent with the SSD-12 by Toussaint et al. (2016).

*Hypothesis 4*: The SSD-12-P is expected to show acceptable reliability and validity close to the original SSD-12 (Toussaint et al., 2016).

(3) Examination of the extent to which parental symptom stress (measured by SSD-12) explain variance in parental stress that is specifically elicited by the physical symptoms of their children (measured by SSD-12-P).

*Hypothesis 5*: Somatic stress, as measured by the SSD-12 in relation to one’s own symptoms, explains a significant proportion of the variance in parent-reported stress related to their child’s symptoms (SSD-12-P). It is assumed that the affective component (worry) in particular is a significant predictor.

## 2 Method

### 2.1 Samples

Our replication sample (study part 1) of adult participants consisted of *N* = 564 individuals (75.5% female; *M* = 44.8 years; *SD* = 14.5). 95.6% indicated Germany as their country of origin, and 72.2% had obtained a high school diploma.

Our validation sample of parents (study part 2) comprised *N* = 144, drawn from the previously described adult sample (*N* = 564). The inclusion criteria for this subsample were: (1) self-identification as a parent, (2) living in the same household as their child(ren), and (3) completion of all items of the SSD-12-P. The mean age of the parent sample was *M* = 42.8 years (*SD* = 7.82) and 86.1% (*n* = 124) were female. A total of 99.3% of the participants reported Germany as their country of origin and 75.7% had high school diploma (‘Abitur’). Children of the recruited parents were *M* = 11.5 years old (*SD* = 5.71). 56.2% (*N* = 82) of the children were female.

### 2.2 Design and procedure

The data were collected within the framework of a multi-wave, large-scale online longitudinal study with the online service soscisurvey. The survey period was 24 months, during which enrolled participants were surveyed every 6 weeks. In order to recruit as many participants as possible, new participants were recruited with each survey wave. Participants were recruited via email lists, social media (e.g., Twitter, Facebook), press releases, and flyers.

In order to recruit a larger number of people with mental health conditions, the study was promoted at the outpatient clinic (notice posted in the waiting area, distribution of flyers). It was clearly communicated that participation in the survey to be entirely voluntary and that it is in no way connected to therapeutic services or therapeutic care. Individuals interested in participating clicked on the survey link, where they were informed about the study objectives and eligibility requirements. If they remained interested, they entered their e-mail address and received a link to the actual survey in their e-mail inbox (double opt-in procedure). The e-mail addresses were stored in the soscisurvey service and were not accessible to the study coordinators. Over a period of 24 months, survey links for the follow-up surveys were sent out every 6 weeks to the participants. Each e-mail contained an opt-out link to cancel receiving additional e-mails.

For the current analysis, we used data from one measurement point (between August 31 and September 27, 2022) during which participants were informed about the validation of an additional parental questionnaire. After receiving detailed information and providing informed consent, they completed all questionnaires (see below). Participants did not receive any compensation for their participation. Before the study began, a positive vote was obtained from the local ethics committee of the psychology institute.

### 2.3 Instruments and questionnaires

The *Somatic Symptom Disorder-B Criteria Scale* (SSD-12) for adults is a 12 item self-report questionnaire with 3 dimensions regarding somatic symptoms: behavioral (1), cognitive (2), and affective (3) components (e.g., ‘Do you worry a lot about having a serious illness?’). In the SSD-12 questionnaire respondents were asked to rate how frequently they experienced each thought, feeling, or behavior on a 5-point Likert scale ranging from ‘never’ (0), ‘rarely’ (1), ‘sometimes’ (2), ‘often’ (3) to ‘very often’ (4). The internal consistency of the total scale was *α* = 0.95 (Toussaint et al., 2016).

The *Somatic Symptom Disorder-B Criteria Scale* parent version SSD-12-P measures the extent to which parents are burdened by their child’s somatic symptoms (e.g., ‘I think that my child’s physical complaints are signs of a serious illness’). In contrast to the original SSD-12, which refers to the respondent’s own somatic symptoms, the SSD-12-P assesses whether analogous SSD-related B-criteria are elicited by somatic phenomena in the respondent’s child.

The mapping of the items to the criteria ‘cognitive’, ‘affective’ and ‘behavioral’ and how the items map from SSD-12 to SSD-12-P are shown in Table 1. The response Likert-scale was adopted identically to the original version of SSD-12. The formulations were first tested and piloted by Jungmann and Witthöft (2020). They tested *N* = 78 parents with the formulations of SSD-12-P. They asked the parents regarding their cognitions, behaviors and affects related to their children’s bodily reactions. The adaptation transitioned the SSD-12 from a patient self-report to a caregiver proxy-report (SSD-12-P) by splitting the locus of physiological symptoms from the locus of psychological distress, prioritizing conceptual equivalence over strict literal translation where necessary. E. g. the original SSD-12 (example is translated from German to English): ‘I worry that my physical symptoms will never go away’ is in SSD-12-P: ‘I worry that my child’s physical symptoms will never go away’. The internal consistency of the pilot-test from of Jungmann and Witthöft (2020) for the total scale was *α* = 0.85, for the affective subscale it was *α* = 0.74, for behavioral *α* = 0.88 and for the cognitive was *α* = 0.5. Following the good results of the pilot test, the wording of the items was adopted in this validation study.

**Table 1:** Item mapping table (SSD12 → SSD-12-P).

Cognitive	“Ich denke, dass meine körperlichen Beschwerden Anzeichen einer ernsthaften Erkrankung sind.” → “Ich denke, dass die körperlichen Beschwerden meines Kindes Anzeichen einer ernsthaften Erkrankung sind.” (Item 1) “Ich bin von der Ernsthaftigkeit meiner körperlichen Beschwerden überzeugt.” → “Ich bin von der Ernsthaftigkeit der körperlichen Beschwerden meines Kindes überzeugt.” (Item 4) “Andere sagen mir, dass meine körperlichen Beschwerden nicht schlimm sind.”→ “Andere sagen mir, dass die körperlichen Beschwerden meines Kindes nicht schlimm sind.” (Item 7) “Ich denke, dass die Ärzte meine körperlichen Beschwerden nicht ernst nehmen.” → “Ich denke, dass die Ärzte die körperlichen Beschwerden meines Kindes nicht ernst nehmen.” (Item 10)
Affective	“Ich mache mir große Sorgen um meine Gesundheit.” → “Ich mache mir große Sorgen um die Gesundheit meines Kindes.” (Item 2) “Meine körperlichen Beschwerden machen mir Angst.” → “Die körperlichen Beschwerden meines Kindes machen mir Angst.” (Item 5) “Ich mache mir Sorgen, dass meine körperlichen Beschwerden niemals aufhören werden.” → “Ich mache mir Sorgen, dass die körperlichen Beschwerden meines Kindes niemals aufhören werden.” (Item 8) “Ich mache mir Sorgen auch in Zukunft durch meine körperlichen Beschwerden beeinträchtigt zu bleiben.” → “Ich mache mir Sorgen auch in Zukunft durch die körperlichen Beschwerden meines Kindes beeinträchtigt zu bleiben.” (Item 11)
Behavioral	“Meine gesundheitlichen Sorgen behindern mich im Alltag.” → “Die gesundheitlichen Sorgen um mein Kind behindern mich im Alltag.” (Item 3) “Meine körperlichen Beschwerden beschäftigen mich den größten Teil des Tages.” → “Die körperlichen Beschwerden meines Kindes beschäftigen mich den größten Teil des Tages.” (Item 6) “Die Sorgen um meine Gesundheit rauben mir Energie.” → “Die Sorgen um die Gesundheit meines Kindes rauben mir Energie.” (Item 9) “Durch meine körperlichen Beschwerden kann ich mich schlecht auf andere Dinge konzentrieren.” → “Durch die körperlichen Beschwerden meines Kindes kann ich mich schlecht auf andere Dinge konzentrieren.” (Item 12)

The *Whiteley Index-7* (WI-7) measures health anxiety and associated cognitive-emotional characteristics in a seven-item based short version (e.g., ‘Do you worry a lot about the possibility that you have a serious illness?’). The 5-point Likert scale range from 1 = ‘not at all’ to 5 = ‘very much’. Cronbach’s alpha was *α* = 0.79 (Glöckner-Rist et al., 2007).

The *PANAS* is a 20-item based questionnaire assessing positive and negative affect (e.g., ‘interested’; ‘nervous’). The 5-point Likert-scale ranges from 1 = ‘very slightly or not at all’ to 5 = ‘extremely’. The internal consistencies were *α* = 0.86–0.90 for positive affect and for negative affect 0.84–0.87 (Glöckner-Rist et al., 2007; Krohne et al., 1996; Watson et al., 1988).

The *PHQ-Stress module* (Berth, 2003; Petrowski et al., 2024) is a 10-items self-report questionnaire assessing psychosocial stress factors potentially related to psychological disorders. Respondents rate how much they are impaired by certain stressors on a 3-point Likert-scale from ‘not at all impaired’ (0) to ‘severely impaired’ (2). The internal consistency in our study was acceptable (*ω* = 0.78). Unfortunately, results could be found in the literature for the PHQ, but not explicitly for the PHQ-Stress module.

The *Somatic Symptom Scale-8* (SSS8) from Gierk et al. (2014) is a questionnaire to measure the extent of somatic burden of the previous 7 days. It consists of eight items and was measured using a five-point likert scale (from “not at all” to “very much”). The internal consistencies were *α* = 0.81.

### 2.4 Statistical analyses

The hypotheses 1 and 3 (replication of the three-factor structure and find the same structure in the parental questionnaire) were tested in a confirmatory factor analysis. To test reliability and validity of both questionnaires, we calculated Mc Donald’s Omega and Cronbach’s Alpha as well as a correlation matrix to test for structure, convergent and discriminate validity. To examine whether and if, the extent to which parental symptom stress (measured by SSD-12) explain variance in parental stress by their children’s symptoms, we calculated a regression analysis. We also tested further relevant predictors by multiple regression analysis.

Both confirmatory factor analyses were performed using the lavaan package in R (Rosseel, 2012). Visualizations of the model structure were created with the sem package (Hayfield and Racine, 2008) and refined using semPlot and semTools (Grubinger et al., 2014; Rosseel, 2012). Internal consistencies as reliability indices were calculated using the psych package (Schmidberger et al., 2009). Correlation and regression analyses were conducted using base R functions from the stats package. Correlation matrices were visualized with the corrplot package (Wei and Simko, 2010).

## 3 Results

**Figure 1:**
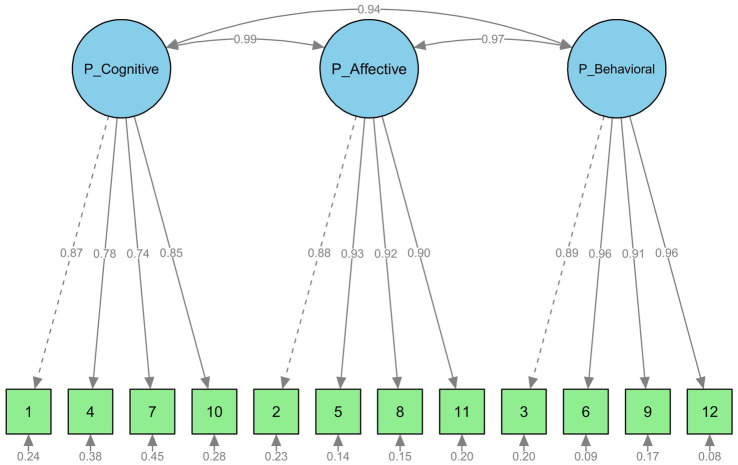
SSD-12-P: Confirmatory factor analysis of parent dataset.

**Figure 2:**
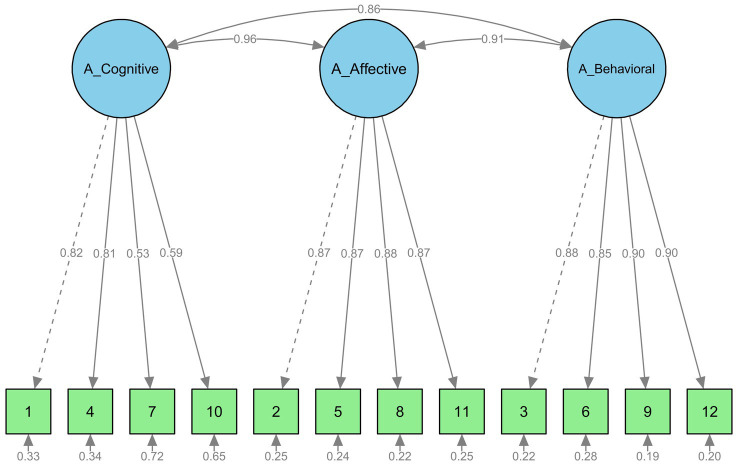
SSD-12: Confirmatory factor analysis of adult dataset.

For each questionnaire confirmatory factor analyses (for SSD-12 and SSD-12-P) were conducted based on the three-factor structure of the SSD-12 reported in the literature (Toussaint et al., 2016). Table 2 summarizes the model fit indices for the three-factor structure: (1) of the original validation model reported by Toussaint et al. (2016), included to allow a direct comparison with the present analyses, (2) our replication of this adult model, and (3) the new parent version evaluated in this study (SSD-12-P). The fit indices were comparable to, or slightly lower than, those reported by Toussaint et al. (2016). In addition, chi-square difference tests indicated that the three-factor model fit the data significantly better than a one-factor model. Figures 1, 2 illustrate the standardized estimates of the three-factor Confirmatory Factor Analysis models for the SSD-12-P and SSD-12. Factor intercorrelations demonstrate high convergence, ranging from 0.94 to 0.99 in the parent model and 0.86 to 0.96 in the adult model. The SSD-12-P items show robust standardized factor loadings between 0.74 and 0.96, structurally comparable to the adult SSD-12 replication showing loadings between 0.53 and 0.90. Figures 1, 2 show that all factor loadings in both models are acceptable or good (≥0.4). The SSD-12-P items (Figure 1), reformulated to index parental stress through children’s symptoms show factor loadings comparable to those of the original SSD-12 items (see the replication in Figure 2).

**Table 2:** Psychometric properties and model characteristics.

Measure/model	SSD-12 original (Toussaint et al., 2016)	SSD-12 replication (adults)	SSD-12 parents (validation)
Sample size	*N* = 698	*N* = 557	*N* = 147
Sample description	Adult Psychosomatic outpatients	Adults	Parents of the *N* = 557 SSD-12 Replication-Sample (community sample)
Internal consistency (*α*; McDonald’s *ω*)	0.94; not reported	0.95; 0.97	0.97; 0.98
1 factor model	CFI: 0.99 TLI: 0.98 RMSEA: 0.09	CFI: 0.9 TLI: 0.88 RMSEA: 0.14	CFI: 0.95 TLI: 0.94 RMSEA: 0.12
3 factor model	CFI: >0.99 TLI: >0.99 RMSEA: 0.06	CFI: 0.94 TLI: 0.93 RMSEA: 0.11	CFI: 0.97 TLI: 0.96 RMSEA: 0.09
Model preference	3-factorial theoretically preferred, 1 factor also acceptable	3-factor better than 1 factor	3 factor better than 1 factor
Chi-Square-difference test	Not reported	3-factor significant improved	3-factor significant improved

Reliability of the SSD-12 and SSD-12-P: Both questionnaires demonstrate very high internal consistencies (see Table 2).

Validity of the SSD-12 and SSD-12-P: Table 3 shows the results of the correlations of all questionnaires. With regard to validity, the SSD-12 showed particularly strong correlations with the WI-7, PANAS negative affect, as well as with PHQ-Stress, and a small negative correlation with PANAS positive affect. For the SSD-12-P, especially strong correlations were observed with negative affect, the WI-7, and PHQ-Stress. As well, there was a small negative correlation with PANAS positive affect.

**Table 3:** Correlation matrix.

	1. SSD-12-P	2. SSD-12	3. WI-7	4. PANAS_pos	5. PANAS_neg	6. PHQ-Stress
1. SSD-12-P						
2. SSD-12	0.585					
3. WI-7	0.495	0.815				
4. PANAS_pos	−0.131	−0.237	−0.217			
5. PANAS_neg	0.430	0.482	0.408	−0.240		
6. PHQ-Stress	0.526	0.577	0.479	−0.340	0.579	0.609

SSD-12-P: Somatic Symptom Disorder - B Criteria Scale for Parents.SSD-12: Somatic Symptom Disorder - B Criteria Scale.WI-7: Whiteley Index-7.PANAS: Positive and Negative Affect Schedule.PHQ-Stress: Stress item of the Patient Health Questionnaire.

Regression analysis: SSD-12, PANAS negative affect, PANAS positive affect, SSS8, PHQ-Stress and WI-7 explain together *R*^2^ = 6.81% of SSD-12-P. The only high significant variance explanation is shown by SSD-12 (SSD-12: *β* = 0.32, *z =* 2.67, *p* = 0.008). None of the other predictors were significant (PANAS_neg: *β* = −0.08, *z* = −0.32, *p* = 0.75; PANAS_pos: *β* = −0.07, *z* = −0.32, *p* = 0.37; SSS8: *β* = −0.09, *z* = 0.44, *p* = 0.44; WI-7: *β* = −0.20, *z* = −0.42, *p* = 0.68; PHQ-Stress: *β* = 0.37, *z* = 1.96, *p* = 0.05).

## 4 Discussion

The present study aimed (1) to replicate the established three-factor structure and psychometric characteristics of the SSD-12 in an adult sample and (2) to extend this work by validating a new parent version (SSD-12-P) assessing parental distress attributable to children’s somatic symptoms.

Summarizing, findings support the proposed three-factor solution in both questionnaires (SSD-12 and SSD-12-P) and provide evidence for very good reliability as well as convergent validity, including the expected pattern of associations with related constructs. With respect to model fit, CFI and TLI values were consistently good for both the adult and parent versions, whereas RMSEA values were comparatively higher (indicating a somewhat poorer model fit).

To account for potential deviations from normality in RMSEA of SSD-12 and SSD-12-P, we therefore estimated models using robust maximum likelihood (MLM) with the Satorra-Bentler correction (Table 2); the overall pattern of fit remained largely unchanged. Higher RMSEA values can occur in CFA models with limited degrees of freedom and may reflect localized misfit; therefore, RMSEA was interpreted alongside the other fit indices (Kenny et al., 2015). Given the very high intercorrelations among the three factors, we also evaluated one-factor alternatives. However, chi-square difference tests indicated that the three-factor model fit significantly better than the one-factor model for both the SSD-12 and the SSD-12-P, and the overall pattern of fit indices consistently favored the three-factor solutions (Toussaint et al., 2016). Despite the strong overlap between dimensions, retaining the three sub facets is consistent with the conceptualization of the SSD B-criteria and may be useful for differentiating domains in research and clinical practice (Hoheisel et al., 2024).

Finally, model fit for the parent version was generally stronger than for the adult SSD-12 replication. Although the three factors were highly intercorrelated, the SSD B-criterion is conceptually multidimensional, comprising cognitive (e.g., symptom-related beliefs and catastrophizing), affective (e.g., health-related anxiety), and behavioral (e.g., excessive checking or avoidance) responses to somatic symptoms. Thus, retaining the three sub facets may be useful because it allows a more fine-grained characterization of symptom-related distress and offers differentiated targets for research and clinical interventions beyond a purely unidimensional total score. Although the three-factor model showed the best fit, the high inter-factor correlations indicate substantial overlap among the dimensions and suggest a strong common component, but the present study was not intended to identify an empirically optimal factor structure in an exploratory manner. Instead, the instrument is explicitly designed for practical use in clinical settings, aiming to directly operationalize the DSM-5 conceptualization of the B criterion across cognitive, affective, and behavioral manifestations. From this applied perspective, the three-factor solution is conceptually necessary, even if a considerable proportion of variance is shared across the subscales. While the total score serves as an indicator of overall B-criterion severity, the distinct subscale scores provide the critical information required in daily practice to establish a differentiated diagnosis, formulate a case conceptualization, and derive targeted therapeutic interventions.

In Table 3 the construct validity of SSD-12 is replicated well (e. g. WI-7: *r* = 0.71 Toussaint et al., 2016; WI-7: *r* = 0.82). Findings indicate further that higher parental somatic distress related to their child’s symptoms (SSD-12-P) is associated with parents’ own symptom related distress (SSD-12) and health anxiety, supporting convergent validity (SSD-12: *r* = 0.59; WI: *r* = 0.50). In contrast, SSD-12-P showed only a small negative association with positive affect (PANAS_pos: *r* = −0.13), which is consistent with discriminant validity. At the same time, SSD-12-P correlated moderately with negative affect (PANAS_neg: *r* = 0.43) suggesting that parental somatic distress is meaningfully related to general negative emotionality but not reducible to it. This pattern is plausible given that negative affect can heighten attention to bodily sensations and symptom reporting, particularly among individuals experiencing greater distress in response to somatic symptoms. Furthermore negative affected people are led to report more symptoms (Petzke et al., 2024).

The multiple regression analysis revealed that SSD-12, PANAS, SSS-8, PHQ-Stress, and WI-7 together explained only 6.81% of variance in SSD-12-P (Table 3), with SSD-12 as the sole significant predictor (*β* = 0.32, *p* = 0.008). This modest effect size aligns with the instruments distinct yet related constructs: SSD-12 and SSD-12-P both tap DSM-5 B-criteria but reference parents own versus child-triggered symptoms in the same individuals, limiting unique predictive overlap. Non-significant predictors (e.g., PANAS_neg *β* = −0.08 despite *r* = 0.43; WI-7 *β* = −0.20 due to multicollinearity with SSD-12, *r = *0.82) further support discriminant validity, underscoring the robustness of SSD-12-Ps, without implying strong intergenerational transmission. It highlights the targeted proxy assessment utility.

Parents experiencing significant somatic distress may be more likely to develop health anxiety by proxy (Ingeman et al., 2024), which could increase vigilance and emotional reactivity toward their children’s bodily complaints. Such heightened monitoring and concern might, in turn, contribute to the maintenance of symptoms and potentially to chronicity. In line with this, previous research suggests that parental somatization can be an important predictor of children’s emotional functioning (Jungmann et al., 2022), underscoring the value of considering familial emotional-somatic dynamics in both research and clinical practice. These findings also point to two implications. First, particularly for internalizing symptoms, obtaining self-reports from children and adolescents appears important because parental ratings may partly reflect parents’ own symptom experiences and illness-related concerns. Second, the results are broadly consistent with interoceptive predictive coding accounts, which propose that symptom perception—including perceptions of others’ symptoms—can be shaped by prior experiences (‘priors’). Accordingly, parents with higher bodily symptom burden or illness anxiety may have a greater tendency to notice, interpret, and worry about somatic sensations in their children (Jungmann et al., 2022; Ingeman et al., 2024; Van den Bergh et al., 2017).

Theoretically, replicating the SSD-12 structure reinforces the underlying conceptualization of SSD B-criteria as comprising cognitive, affective, and behavioral responses rather than a purely undifferentiated distress factor, supporting their use as separable (though strongly related) facets in etiological models. Practically, validating the SSD-12-P adds a targeted parent self-report that captures parental distress attributable to the child’s somatic symptoms, complementing child symptom measures and enabling a more family-centered assessment. This facilitates identifying clinically relevant parental response patterns (e.g., heightened worry or reassurance-seeking) that may be important intervention targets alongside the child’s symptoms. Importantly, SSD-12-P scores should not be interpreted as reflecting general parental burden or everyday concern about a child’s health. Rather, higher scores indicate a by-proxy manifestation of SSD-related B-criteria in the parent, namely maladaptive symptom-focused cognitions, affective distress, and behavioral responses triggered by the child’s bodily symptoms.

## 5 Limitations

This study relied on self-report data, which may be affected by reporting biases and shared method variance, and may have inflated associations between parental and child somatic measures. In addition, the cross-sectional design precludes causal conclusions; longitudinal studies are needed to clarify temporal ordering and potential (bi)directional links between parental and child somatic symptom distress. This cross-sectional limitation strongly precludes causal or directional inferences. The relatively homogeneous sample further limits the generalizability of our findings to more diverse populations and cultural contexts. Finally, data were collected during the COVID-19 pandemic, which may have influenced participants’ symptom perception and reporting (e.g., possibly heightened vigilance or concern) (Wierenga et al., 2021).

## 6 Future directions

Future research should employ longitudinal designs to clarify the developmental trajectories and (bi-)directional links between parental somatic burden and children’s emotional and somatic symptoms. Studies that combine questionnaire data with physiological and observational indicators of early parent–child synchrony could deepen the understanding of mechanisms through which symptom-related distress is transmitted or maintained within families. From an applied perspective, it would be valuable to develop and evaluate interventions that address parental somatic distress and health anxiety by proxy alongside children’s emotion regulation to potentially reduce intergenerational risk. Cross-cultural research is also needed to test generalizability and to identify culturally specific patterns in familial SSD-related processes. Finally, further refinement of measurement approaches—including efforts to better differentiate SSD dimensions and the routine inclusion of child self-report in addition to parent ratings—may provide more complete and clinically informative assessments.

## 7 Conclusion

Overall, the replicated factor structure and evidence for reliability and validity indicate that the SSD-12 yields stable measurement properties across samples, supporting its use for screening and routine outcome monitoring in adult care. The newly developed parent version SSD-12-P likewise demonstrated strong psychometric performance (factorial validity, internal consistency, convergent/discriminant validity), indicating robust measurement for assessing parental distress related to children’s somatic symptoms. Together, these instruments provide a useful basis for capturing parental responses and coping patterns, advancing research on family dynamics in somatic symptom presentations, and guiding child- and parent-focused interventions. Thus, the SSD-12-P is a valuable instrument in all settings where work is done with burdened parents, such as psychiatric clinics, parent–child facilities, and counseling centers.

## Data Availability

The raw data supporting the conclusions of this article will be made available by the authors, without undue reservation.
